# Modelling spatially realistic local field potentials in spiking neural networks using the VERTEX simulation tool

**DOI:** 10.1186/1471-2202-15-S1-P130

**Published:** 2014-07-21

**Authors:** Richard J Tomsett, Matt Ainsworth, Alexander Thiele, Mehdi Sanayei, Xing Chen, Alwin Gieselmann, Miles A Whittington, Mark O Cunningham, Marcus Kaiser

**Affiliations:** 1 School of Computing Science, Newcastle University, NE1 7RU, UK; 2 Institute of Ageing and Health, Newcastle University, NE4 5PL, UK; 3 Hull York Medical School, University of York, YO10 5DD, UK; 4 Institute of Neuroscience, Newcastle University, NE2 4HH, UK

## 

Local field potentials (LFPs), measured using extracellular electrodes, provide information about local neuronal population activity. The development of multi-electrode arrays (MEAs) that measure LFPs simultaneously at different network locations has led to a renewed interest in the LFP as a measure of neuronal network activity [1]. While there is a well-understood relationship between neuronal current-sources and LFPs, the complexities of current generation in spatially organised neuronal networks mean that relating LFP measurements to the underlying network dynamics is non-trivial [1]. Forward modelling studies have shown that the use of multicompartment models incorporating dendritic structure is crucial for simulating spatially realistic LFPs [2]. However, most forward models of neural network dynamics use simplified single-compartment neurons, because they are easier to parameterise and much less computationally expensive to simulate.

Here, we describe two contributions to the effort to understand LFP generation in neuronal networks. First, we investigated reduced compartmental models to assess their suitability for producing realistic spatial LFP characteristics. We show that the chosen reduction method [3] creates compartmental models that preserve essential spatial LFP features, while being computationally cheap enough for large network simulations. Second, we developed the Virtual Electrode Recording Tool for EXtracellular potentials (VERTEX): a tool for simulating LFPs in spatially organised, spiking neural networks. We implemented VERTEX in Matlab (Mathworks Inc., Natick, MA, USA) as it is widely used throughout the neuroscience community, and we provide a simple interface based on parameter specification so that VERTEX can be used even by non-programmers. Code vectorisation and parallelisation methods allow VERTEX to simulate networks containing hundreds of thousands of reduced compartmental neurons in reasonable time. We demonstrate how VERTEX can be used to simulate LFPs at hundreds of locations in a large-scale, spatially organised cortical network model, and how these results can be used in conjunction with experimental data, by comparing simulation results with MEA recordings of persistent gamma oscillations made in macaque neocortical tissue *in vitro*.
